# Public service motivation and perceived community resilience: sense of community and sense of community responsibility as mediators

**DOI:** 10.3389/fpsyg.2025.1614637

**Published:** 2025-11-19

**Authors:** Jinmin Niu, HongJie Dong, ShiXiang Liu

**Affiliations:** 1 School of Psychology, Inner Mongolia Normal University, Hohhot, China; 2 Teacher's College of Beijing Union University, Beijing, China

**Keywords:** public service motivation, sense of community, sense of community responsibility, perceived community resilience, community resilience

## Abstract

**Objective:**

Perceived community resilience (PCR) serves as the foundation for sustainable development, yet the psychological impetus driving the enhancement of PCR remains to be elucidated. This study delved into the psychological motivation underpinning PCR by examining the interrelationships between PCR and public service motivation (PSM), sense of community (SOC), and sense of community responsibility (SOC-R).

**Methods:**

A total of 550 participants from three cities in China were tested using the Public Service Motivation Intercultural Scale, the Sense of Community Responsibility Scale, the Sense of Community Scale, and the 10-Item Conjoint Community Resiliency Assessment Measure.

**Results:**

There was a significant positive correlation between PSM, SOC-R, SOC, and PCR scores; (2) PSM had a significant and direct effect on PCR; (3) PSM had a significant indirect effect on PCR through the chain mediators of SOC-R and SOC.

**Conclusion:**

These findings clarify the psychological motivations underlying PCR and could inform strategies for improving community sustainability.

## 1 Introduction

Communities around the world have long been exposed to a wide range of disasters, meaning “a serious disruption of the functioning of a community or society involving a wide range of human, material, economic or environmental losses and impacts beyond the ability of the affected community or society to cope with with its own resources” (United Nations International Strategy for Disaster Reduction, 2009). The ability of communities to withstand shocks relies on community resilience, which also forms the basis of regional resilience and urban resilience (Yang et al., 2020a). The term Community Resilience (CR) has been used to describe the ability of communities to function and respond in crisis environments of continuous change and uncertainty (Maclean et al., 2014); or to respond creatively to crises and to allow communities to recover from the risk of disruptive impacts (Rochira et al., 2023).

Researchers have long noted the important role of community resilience in post-disaster recovery and environmental risk reduction. However, CR measurement based on objective indicators failed to reach a consensus due to the diverse economic, social organization and geographic settings of communities. As a result, researchers have gradually begun to shift their perspective to residents' perceived community resilience (Pfefferbaum et al., 2015; Yang et al., 2020a). Residents' perceptions of CR(PCR) not only reveal how communities use resources and collaborate to overcome adversity (Chaskin, 2008), but also go some way to enhancing the wellbeing and security of community residents (Suleimany et al., 2022). Drawing on social capital theory, Leykin et al. (2013) considered leadership, collective efficacy, preparedness, place attachment and social trust as residents' assessments of the five constructs of PCR.

Residents' assessments of CR are closely related to the social capital that a community possesses, and the adequacy of the stock of social capital is critical to a community's ability to cope with risk or residents' assessments of its capacity (Lee, 2020). Communities with higher levels of CR are better able to adapt and recover when risks occur. The formation of social capital can assist in strengthening this cohesion in recovery (Behera, 2023). However, social capital is not generated directly by the community; rather, it requires individuals within the community to contribute to or participate in the community group (Coleman, 1988). In other words, communities require individuals who are prepared to contribute to the creation of social capital in order to enhance their resilience. In this regard, public service motivation (PSM), defined as a behavioral tendency for individuals motivated by a desire to contribute to the public good, help others, or improve society (Gans-Morse et al., 2022), has been identified as an effective predictor of volunteering behavior and willingness to participate (Holt and Piatak, 2023). While PSM originated in public administration research to understand motivation in public sector contexts, its core elements—such as commitment to public values, compassion, and self-sacrifice—represent generalizable pro-social dispositions relevant to any collective setting where contributions to the common good are needed (Brewer, 2003; Nowell et al., 2016). Thus, in neighborhood or community contexts, PSM can be understood as a foundational motivational driver that predisposes individuals to engage in behaviors that build and sustain community social capital. This motivation plays a role in the accumulation of social capital in the community and may also encourage individuals to engage in behaviors that are beneficial to the community, thereby increasing residents' PCR. While this inference has not been confirmed in community research, there is evidence of this potential role for PSM (Esteve et al., 2016).

Accordingly, this study will explore and validate the use of PSM to explain residents' PCR based on social capital theory. In this way, it will explore the psychological pathway from micro-individual perspective to meso-level PCR and propose appropriate improvement strategies.

### 1.1 Perceived community resilience and community resilience

CR is an important theoretical outcome of the application of resilience theory to the practice of community disaster prevention and mitigation, and previous studies have generally agreed that it has a three-dimensional meaning of “capacity-process-outcome” (Wolke et al., 2025): (1) capacity dimension, refers to the collection of community's ability to resist disasters in the process of disaster, including stabilization, resilience and adaptive capacity; (2) process dimension, regards resilience as the whole process of improving the adaptive capacity of the system and ultimately adapting to the disaster, which refers to not only the specific process of adapting to a particular disaster, but also the cyclic process of improving the adaptive capacity of the system; (3) outcome dimension, resilience is one of the goals that the community's strategy of preventing and mitigating disasters is attempting to achieve, and it can be used as a test for preventing and mitigating disaster.

However, there is no universally accepted objective measure of community resilience. One reason for this is that, as Halekotte et al. (2025) questioned when sorting through three understandings of resilience, “Do we practice what we preach?”, finding that there are understandings of resilience that contradict measurements in a large number of previous studies (Bruneau et al., 2003; Ouyang and Wang, 2015; Shafieezadeh and Burden, 2014). On the other hand, because communities vary in terms of their economy, social organization, and geography, most measurements of community resilience in previous studies have focused on single disasters or specific communities (Joerin et al., 2012; Zhong et al., 2020; Ainuddin and Routray, 2012). This greatly increases the generalisability of CR measures and the measurement of differences in resilience between communities.

PCR is publics' belief in their community's ability to withstand and recover from disaster crisis (Zhang and Shay, 2019). This individual perspective offers unique “bottom-up” insights into how individual experience and communication fosters or impedes resilience building (Houston et al., 2017) thus allowing for “community-driven interventions” (Pfefferbaum et al., 2015), easurements can also be made to fit most communities (Leykin et al., 2013). Pfefferbaum et al. (2013) designed the “communities advancing resilience toolkit” (CART) as the first instrument to measure PCR. Subsequently, in the context of social unrest under the threat of missiles and terrorism, Israeli researchers reconsidered the CART and designed the 28-item “conjoint community resiliency assessment measure” (CCRAM) to study community resilience under social uncertainty (Leykin et al., 2013). A simplified version of the 10-item CCRAM has shown high acceptance in many countries in follow-up studies (Eshel and Kimhi, 2016; Cui and Han, 2019). While perceived community resilience cannot truly determine resilience until a disaster occurs, if perceived community resilience is strengthened, it at least indicates a belief that the community will recover after a disaster.

Regarding the individual-level influences on PCR, previous studies have favored gender and age (Soetanto et al., 2017), length of residence in the community (Redshaw et al., 2018), and perceptions of family and community (Houston et al., 2017). It is undeniable that these factors influence how residents evaluate the resilience of their own communities, but the role of these unchangeable individual characteristics on “how to improve community resilience” is difficult to interpret. At the community level, the challenges posed by natural disasters and changing times have created a need for people who are willing to take responsibility and contribute to the community. This personal motivation to “provide service to others with the intention of doing good for others and society”, i.e., the PSM (Perry and Hondeghem, 2008), also plays an important role in all phases of a community disaster (Moore et al., 2020; Holt and Piatak, 2023).

### 1.2 Public service motivation and perceived community resilience

Nakagawa and Shaw (2004) pioneering concept of public service motivation describes individuals motivated by a desire to contribute to the public good, help others, or improve society (Gans-Morse et al., 2022). However, there is not much research on PSM in community contexts. Established research on PSM focuses on leadership, business or organizational communities (Brincker and Pedersen, 2023; Boyd, 2023), and few studies have been used in residential communities. In light of this, Nowell et al. (2016) have challenged the idea that PSM and community experience would provide complementary perspectives. Emerging research has also found the importance of public service motivation and community responsibility in motivating community action during COVID-19 (Toubøl et al., 2023). On the other hand, combining public service motivation with community experience may also help us to develop a deeper understanding of CR.

PSM was initially used as a theoretical lens to explain public servants‘ service to society (Perry and Wise, 1990), and subsequent scholars began to realize that it was not just a characteristic of public servants, but a behavioral tendency of all individuals (Brewer, 2003). This behavioral tendency can be a valid predictor of an individual's volunteering behavior and willingness to participate (Holt and Piatak, 2023). Although it may promote individuals to increase behaviors that benefit their community thereby increasing residents' ratings of CR (Esteve et al., 2016), this link has not yet been demonstrated in community studies. Clarifying the issue of the relationship between public service motivation and PCR could interpret the endogenous motivation of residents to engage in PCR building and help humanize and pinpoint community building measures.

Additionally research has shown that individuals' public service motivation is strongly correlated with pro-social behavior (Holt and Piatak, 2023), and that pro-social behavior among community residents improves the community's ability to cope with disasters (Tindowen and Bagalayos, 2018) and helps to restore the community's day-to-day functioning (Moore et al., 2020). And public service motivation can also be used to predict the contribution individuals make to the public good (Esteve et al., 2016). Communities, as meso-structures of society, naturally represent the public interest, and then PSM can also be used as a predictor of the contribution individuals make to their communities. On the flip side, an individual's level of motivation affects their cognitive processes, which in turn affects their perception and behavior toward their surroundings (Vroom et al., 2015). It is undeniable that communities are the environments in which their residents reside, and in such community environments, public service motivation, as a form of motivation, it is reasonable to propose that the PSM of community residents will influence their evaluation of CR.

**Figure 1:**
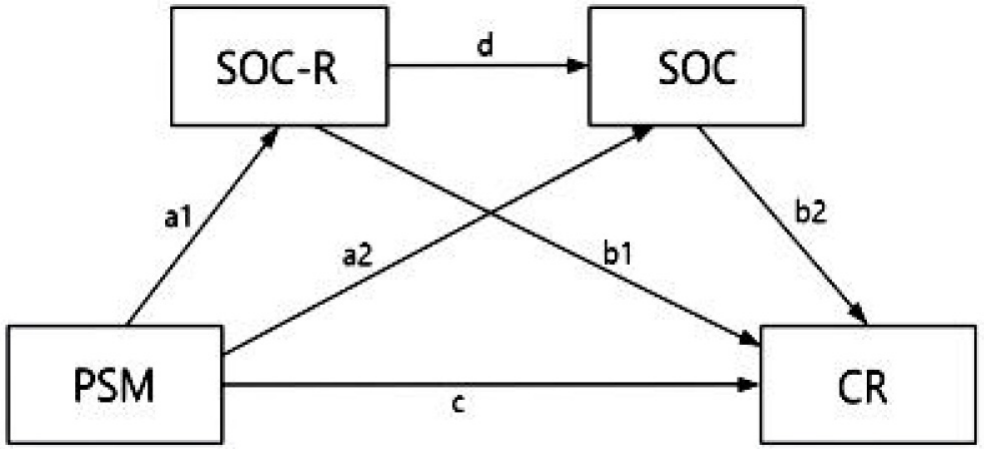
Hypothetical model of the relationships between variables. PSM, Public Service Motivation; SOC-R, Sense of Community Responsibility; SOC, Sense of Community; CR, Community Resilience.

On the basis of these theories and research findings, we hypothesized that:

**H1**: PSM affects perceived CR (c) (Figure 1).

### 1.3 The mediating role of sense of community responsibility and sense of community

Public service motivation is expressed as an individual's values and beliefs about general motivational dispositions toward public service, which can exist independently of a particular context (Nowell et al., 2016). In previous research, Sense of Community Responsibility (SOC-R) and Sense of Community (SOC) share a similar nature and pattern with PSM, both based on the same values and beliefs (Boyd and Nowell, 2020). However, a sense of community responsibility and a sense of community demonstrate a subordination to a specific community context (Francis et al., 2012; Boyd et al., 2018). And it is this subordination that leads to the fact that an individual's SOC-R and SOC will be more closely tied to the community to which the individual belongs. Both are also more likely to have a direct impact on CR as opposed to PSM. And it has also been found in previous research that PSM may, to some extent, translate general motivational tendencies into context-specific identities, a sense of community, and attitudes of responsibility and commitment (Boyd and Nowell, 2020).

The general attribute of PSM exists independently of context, whereas SOC-R reflects an individual's sense of duty to a particular community (Boyd et al., 2018; Nowell et al., 2016). Although the two values belong to different audiences, researchers have found that both are significant predictors of organizational behavior, and that the latter fully mediates the effect of the former on indicators of collaborative leadership (Boyd et al., 2018). Other studies have also shown that there is a clear link between individuals' motivation for public service and the consequent increase in responsibility and commitment to their work (Nowell et al., 2016; Masukela et al., 2023; Chen et al., 2023). That is to say that individuals with high levels of PSM are more likely to have a strong sense of responsibility to a particular community. The same hypothesis has been proposed by Boyd and Nowell (2020) and their findings provide support for this hypothesis, in the context of a one-time community co-operation, where it was found that individuals who reported higher levels of motivation to perform PSM were more likely to report a stronger SOC-R to their community.

Most previous studies have focused on factors such as the type of disaster, its magnitude and the type of community. (Ainuddin and Routray, 2012; Zhong et al., 2020; Valdez et al., 2019; Goidel et al., 2019; Abenayake et al., 2018). Since Aldrich and Meyer (2015) began to turn their lens to the impact of social capital on CR, disaster and resilience research by a growing number of scholars has shown that this social capital in the form of ties, bridges, and links has an important role to play in all phases of disaster occurrence (Frounfelker et al., 2020; Behera, 2023; Nugraha et al., 2024). Social capital describes anything that facilitates individual or collective action arising from relational networks, reciprocity, trust and social norms (Coleman, 1988). The purpose of social capital in communities is to promote community social norms and social networks that are geographically bounded (Hoi et al., 2018). Psychological factors such as sense of community (SOC) and sense of community responsibility (SOC-R) are not social capital *per se*, but rather key psychological precursors or components that facilitate its formation and maintenance (Nowell and Boyd, 2014; Boyd and Nowell, 2020). These psychological senses are foundational because they motivate the very behaviors—such as participation, cooperation, and adherence to norms of reciprocity—that generate the relational networks, trust, and shared resources characterized as social capital (Coleman, 1988; Putnam, 1993). In this way, SOC and SOC-R represent the micro-level psychological underpinnings that are crucial for the emergence of meso-level community social capital, which in turn enhances community resilience (Aldrich and Meyer, 2015).

In our model, we position SOC-R and SOC not as direct proxies for social capital, but as critical mediating psychological mechanisms. They translate the general, context-free motivational orientation of PSM into community-specific affective bonds (SOC) and a sense of duty (SOC-R) (Boyd and Nowell, 2020). It is these context-specific psychological states that are theorized to more directly foster the patterns of interaction, trust, and reciprocity that constitute community social capital, which ultimately bolsters perceived community resilience. There is a strong link between ‘responsibility' and CR, with Colten et al. (2008) suggesting that micro-level personal responsibility is key to building a resilient community, and Mullins and Soetanto (2011) suggesting that macro-level social responsibility has an important impact on community resilience assessment. Based on this, it is reasonable to hypothesize that a SOC-R that is more closely linked to the community also has a positive effect on CR. And on the one hand, the level of an individual's SOC-R also affects his or her perception of the surrounding environment (Wells et al., 2011), and this sensitivity to the environment may affect residents' perceived CR.

In consideration of the aforementioned theoretical and research findings, the following hypothesis is proposed for consideration:

**H2:** Individual PSM has an effect on perceived CR through the mediation of SOC-R (a1 → b1) (Figure 1).

Sense of community was first defined as ‘a structured and stable sense of belonging that is characterized by perceptual expectations and feelings of interdependence similar to those of others and a willingness to maintain them (Sarason, 1974)'. Researchers have explored studies on the relationship between public service motivation and sense of community. The findings revealed that PSM was a significant predictor of SOC and that SOC played a significant mediating role in the prediction of employee wellbeing by PSM (Boyd and Nowell, 2020). In addition, similar findings were obtained in a recent study of organizational citizenship behaviors, where PSM significantly increased sense of community and organizational citizenship behaviors (Jiartana et al., 2023).

SOC has not been well researched in terms of residents' perceptions of CR but Sonn and Fisher (1998) have suggested that SOC may provide new perspectives on viewing CR. A recent study of the COVID-19 also found that SOC was significantly associated with CR (Mannarini et al., 2022). And it has been used as a key predictor variable in previous studies of CR (Patel et al., 2017).

In light of the aforementioned theoretical and research findings, we put forth the following hypothesis:

**H3:** Individual PSM has an effect on perceived CR through the mediation of SOC (a2 → b2) (Figure 1).

### 1.4 Relationship between sense of community responsibility and sense of community

As mentioned above, SOC-R is a community-specific psychological construct with a subordination relationship. The sense of dependence and belonging contained in the SOC are also subordinate, which is what distinguishes it from other psychological constructs (Francis et al., 2012). It has been shown that there is a positive correlation between both them, and that they have much in common as psychological motivators in emotional connectedness, identity, and concern for others (Boyd and Nowell, 2020). And both predict community participation (Boyd and Nowell, 2017; Talò, 2018), civic and political engagement (Prati et al., 2020; Procentese and Gatti, 2019), pro-social behavior (Omoto and Snyder, 2010; Yang et al., 2020b), among others. For their sequential relationship between the two, it has been shown that individuals with a higher sense of responsibility are more likely to have a higher sense of belonging and trust in the group (Nowell and Boyd, 2014). That is, an individual's SOC-R may precede the emergence of a SOC.

On the basis of these research findings, we hypothesized that:

**H4:** Individual PSM has a chain mediating effect on perceived CR through SOC-R and SOC (a1 → d → b2) (Figure 1).

## 2 Materials and methods

### 2.1 Data collection and sample

A total of 550 valid questionnaires were collected by random sampling in Beijing, Wuxi, and Dongguan, China, where the mean age of those surveyed was 44.64 years, with a standard deviation of 9.42. ranging from 30 to 65 years old. Of participants, 33.8% lived in Beijing, 33.1% in Wuxi, and 33.1% in Dongguan. A total of 282 participants (51.3%) were men and 268 (48.7%) were women, and 88.4% of participants were married (and lived with their spouses for more than 6 months in a year). Regarding educational level, 4.2% of participants had junior high school education or below, 18.2% had high school education, 21.3% had junior college education, 51.3% had undergraduate education, and 5.1% had postgraduate education or above.

### 2.2 Measures

The cross-cultural Public Service Motivation Scale as revised by Bao and Li (2016) was used to measure PSM (Kim et al., 2013). This scale comprises eight items; example items are “Meaningful public welfare activities are important to me” and “For me, it is very important to make contributions to social welfare.” All items are rated on a 5-point scale from completely disagree to completely agree (1 = completely disagree; 5 = completely agree). The scale has four dimensions: Attraction to public service, Commitment to public values, Compassion, and Self-sacrifice. The internal consistency of the scale in this study was satisfactory (Cronbach's alpha coefficient: 0.816).

Nowell and Boyd (2014) Sense of Community Responsibility Scale was used to measure SOC-R. The scale comprises six items; All items are evaluated on a five-point Likert scale, ranging from strongly disagree to strongly agree. (1 = strongly disagree; 5 = strongly agree). The main subjects in this study were community residents, so the questionnaire was modified to make it more suitable for the purpose of this study. Some of the modifications include but are not limited to the change of “collaboratives” to “community” in item 1 of the original scale; the change of item 3 “Relative to other collaboratives I've been involved with, I feel a particularly strong sense of responsibility for the success of this collaborative” was changed to “I have a particularly strong sense of responsibility for the success of our community partnership relative to other communities I know”. The internal consistency of the scale in this study was satisfactory (Cronbach's alpha coefficient: 0.864).

SOC was measured using the Sense of Community Scale for Farmers-to-Citizens, developed by Liu and Dong (2019). The scale is applicable to residents of urban communities in China who have a developing SOC. The scale comprises 12 items; example items are “Most of the residents in the community know me” and “I can talk to other residents of the community about the problems I've encountered.” All items are rated on a 7-point scale from completely disagree to completely agree (1 = completely disagree; 7 = completely agree). This scale has three dimensions: Identity Maintenance, Demand Satisfaction, and Development Expectation. The internal consistency of the scale in this study was satisfactory (Cronbach's alpha coefficient: 0.931).

CR was measured using the Leykin et al. (2013) 10-Item Conjoint Community Resiliency Assessment Measure (CCRAM-10), which has been translated and validated by Cui and Han (2019). The CCRAM-10 contains 10 items; example items are “The local government of my community functions well” and “I can count on people in my community to help me in a crisis situation.” The scale has five dimensions: Leadership, Collective Efficacy, Preparedness, Place Attachment, and Social Trust. The internal consistency of the scale in this study was satisfactory (Cronbach's alpha coefficient: 0.916).

### 2.3 Data analysis

SPSS 24.0 statistical methods were employed to determine the averages and standard deviations for the variable data, along with conducting correlation analyses for the primary variables. Then, AMOS 21.0 was used to analyze the validity of each questionnaire and the structural equation model of mediating effect. The assessment of each mediation effect relied on the bootstrapping technique utilizing 5,000 samples to evaluate whether the indirect effects exhibited non-normal sampling distributions (Lockwood and MacKinnon, 1998).

We examined SOC-R and SOC as chain mediators between PSM and CR, as shown in Figure 1. Therefore, three indirect effects of PSM were specified: (1) through SOC-R (a1, b1); (2) through SOC (a2, b2); and (3) through SOC-R and SOC (a1, d, b2). Significant results support the chain mediation model (Hayes, 2018).

## 3 Results

### 3.1 Common method bias test

Since the data were collected using self-report measures, which might lead to common method bias, Harman's single-factor test was conducted to assess common method bias. The results showed that there were 5 factors with eigenvalues greater than 1, and the first factor explained 45.23% of the variance, which is below the critical threshold of 50%. This indicates that there is no serious common method bias in the data of this study (Podsakoff and Organ, 1986).

### 3.2 Correlation analysis of main variables

The descriptive statistics of each variable and the variable intercorrelations are shown in Table 1. The demographic variable of marital status were significantly and positively correlated with PSM (*r* = 0.10, *p* < 0.05), SOC-R (*r* = 0.13, *p* < 0.01), SOC (*r* = 0.11, *p* < 0.05), and CR (*r* = 0.14, *p* < 0.01). Type of residence was significantly and positively correlated with SOC-R (*r* = 0.11, *p* < 0.05), SOC (*r* = 0.12, *p* < 0.05), and CR (*r* = 0.10, *p* < 0.05). PSM was significantly and positively correlated with SOC-R (*r* = 0.75, *p* < 0.001), SOC (*r* = 0.72*, p* < 0.001), and CR (*r* = 0.71, *p* < 0.001). SOC-R was significantly and positively correlated with SOC (*r* = 0.79*, p* < 0.001) and CR (*r* = 0.75, *p* < 0.001). There was also a significant positive correlation between SOC and CR (*r* = 0.85, *p* < 0.001).

**Table 1:** Descriptive statistics (*N* = 550).

**Variables**	**M ± SD**	**PSM**	**SOC-R**	**SOC**	**CR**
PSM	4.18 ± 0.52	1			
SOC-R	4.02 ± 0.68	0.75^***^	1		
SOC	5.41 ± 1.00	0.72^***^	0.79^***^	1	
CR	4.01 ± 0.67	0.71^***^	0.75^***^	0.85^***^	1
Age	46.64 ± 9.42	−0.07	−0.03	−0.03	−0.05
Gender		0.04	0.06	0.05	0.04
Marital status		0.10^*^	0.13^**^	0.11^*^	0.14^**^
Type of residence		0.06	0.11^*^	0.12^*^	0.10^*^

*N* = 550. ^***^p < 0.001,^**^p < 0.01,^*^p < 0.05.

### 3.3 Regression analysis of key variables

The regression analyses of the variables are presented in Table 2. The results show (note: the coefficients below are standardized coefficients) that among the demographic variables, marital status is a predictor of motivation for public service (*b* = 0.10, SE = 0.04, *t* = 2.40, *p* < 0.05), with 95% confidence intervals of [0.02, 0.18]. The other demographic variables were not significant predictors of each predictor variable.

**Table 2:** Results of linear regression analysis for each variable.

**Predictor Variables**		**Age**	**Gender**	**Marital status**	**Type of residence**	**PSM**	**SOC-R**	**SOC**
PSM	Coefficient	−0.07	0.04	0.10^*^	0.08			
	95% CI	[-0.01,0.01]	[-0.05,0.16]	[0.02,0.18]	[-0.01,0.10]			
SOC-R	Coefficient	0.01	0.02	0.04	−0.02	0.38^***^		
	95% CI	[-0.01,0.01]	[-0.05,0.10]	[-0.01,0.10]	[-0.05,0.02]	[0.38,0.55]		
SOC	Coefficient	0.01	0.01	0.01	−0.03	0.29^***^	0.58^***^	
	95% CI	[-0.01,0.01]	[-0.07,0.08]	[-0.06,0.06]	[-0.06,0.02]	[0.27,0.45]	[0.50,0.64]	
CR	Coefficient	−0.03	−0.01	0.04	0.01	0.14^***^	0.13^***^	0.64^***^
	95% CI	[-0.01,0.01]	[-0.08,0.05]	[-0.01,0.10]	[-0.03,0.03]	[0.09,0.26]	[0.05,0.21]	[0.56,0.71]

All coefficients are standardized. CI, Confidence Interval; PSM, Public Service Motivation; SOC-R, Sense of Community Responsibility; SOC, Sense of Community; CR, Community Resilience. ^***^p < 0.001,^**^p < 0.01,^*^p < 0.05.

PSM was positively associated with SOC-R (*b* = 0.40, SE = 0.04, *t* = 10.93, *p* < 0.001, 95% CI [0.38, 0.55]), SOC (*b* = 0.29, SE = 0.05, *t* = 7.70, *p* < 0.001, 95% CI [0.27, 0.45]), and PCR (*b* = 0.14, SE = 0.04, *t* = 4.10, *p* < 0.001, 95% CI [0.09, 0.26]). SOC-R was positively associated with SOC (*b* = 0.58, SE = 0.04, *t* = 15.20, *p* < 0.001, 95% CI [0.50, 0.64]) and PCR (*b* = 0.13, SE = 0.04, *t* = 3.35, *p* < 0.001, 95% CI [0.02, 0.21]). SOC was positively associated with PCR (*b* = 0.64, SE = 0.04, *t* = 17.13, *p* < 0.001, 95% CI [0.56, 0.71]).

### 3.4 Structural equation model

**Figure 2:**
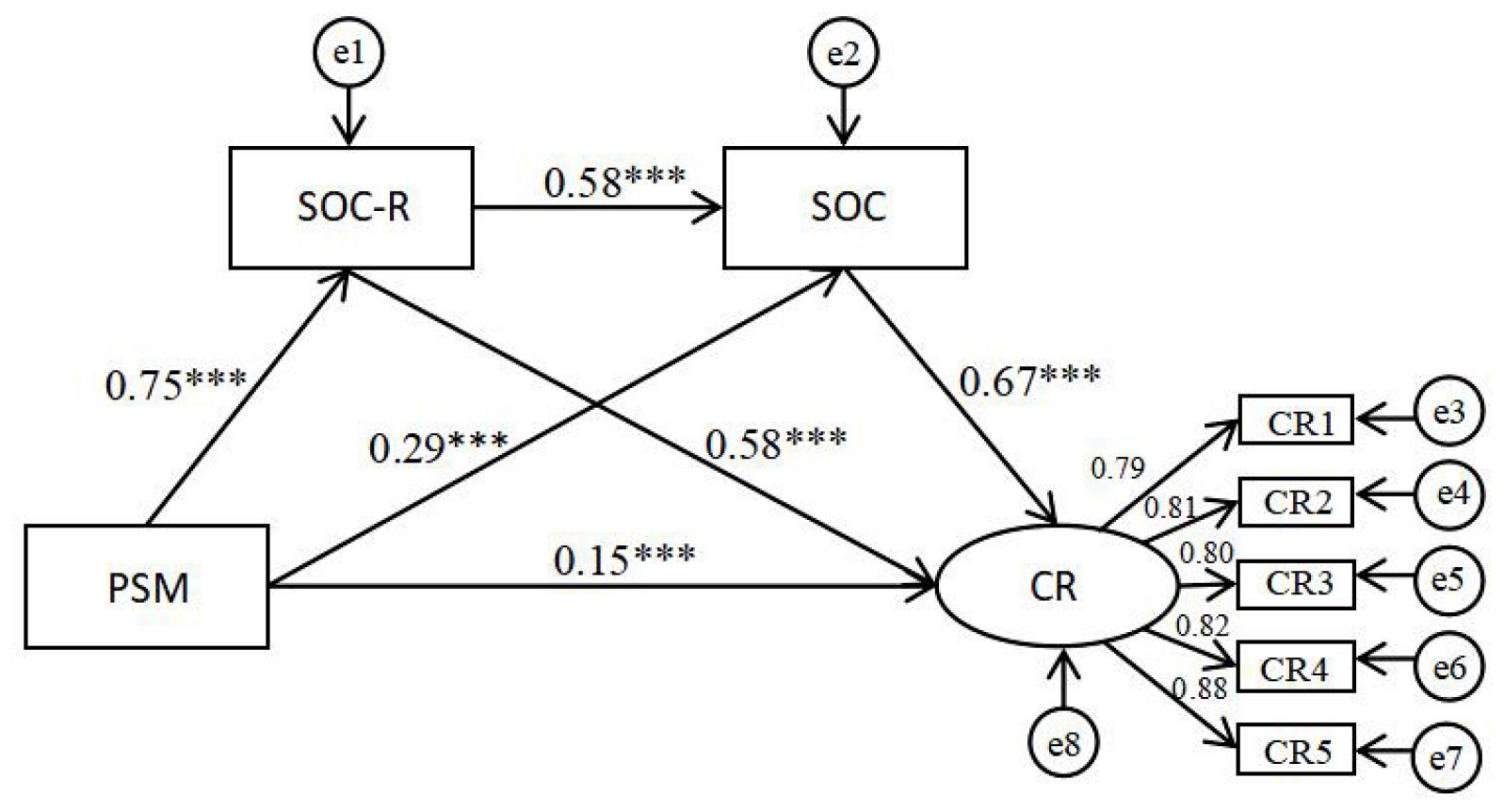
Effect of public service motivation on community resilience through community responsibility and sense of community (standardized coefficients). All coefficients are standardized. CR1, Leadership; CR2, Collective Efficacy; CR3, Preparedness; CR4, Place Attachment; CR5, Social Trust. PSM, Public Service Motivation; SOC-R, Sense of Community Responsibility; SOC, Sense of Community; CR, Community Resilience. ***p < 0.001.

Structural equation modeling was used to validate that community responsibility and sense of community act as chain mediators between public service motivation and community resilience based on the purpose of the study, the constructed model is shown in Figure 2. The fitting results show that the model is good for all indicators (*CMIN/DF*=*2.60, RMSEA* = *0.05 ,CFI* = *0.99, GFI* = *0.98, NFI* = *0.98, TLI* = *0.99*), which suggests that the data fitting validates the theoretical model (Bollen and Long, 1993), and that athe next step of mediation analysis.

Subsequently, a structural equation model was constructed using AMOS 21.0 software for the purpose of testing the hypothetical model. The structural equation model was constructed using a bootstrapping procedure, which entailed the drawing of 5,000 bootstrapping samples and the computation of 95% confidence intervals (95% CI). The results indicated that the total effect of public service motivation on community resilience was significant (*b* = 0.84, SE = 0.05, *p* < 0.001) with a 95% confidence interval of [0.73, 0.94]. More importantly, the total indirect effect of public service motivation on community resilience was also significant (*b*=0.67, BootSE=0.13, 95% BootCI [0.56, 0.81]). The confidence intervals did not contain 0, indicating that the mediating effects of community responsibility and sense of community held (Preacher and Hayes, 2008) and mediated in the model. All indirect effects are shown in Table 3.

**Table 3:** Structural equation model.

**Pathway**	**Hypotheses**	**Coefficient**	**SE**	**95% CI**
PSM → CR	H1(c)	0.15	0.04	[0.03, 0.27]
Total indirect effect		0.92	0.13	[0.56, 0.81]
PSM → SOC-R → CR	H2(a1 → b1)	0.44	0.05	[0.03, 0.23]
PSM → SOC → CR	H3(a2 → b2)	0.19	0.04	[0.15, 0.30]
PSM → SOC-R → SOC → CR	H4(a1 → d → b2)	0.29	0.05	[0.24, 0.46]

All coefficients are standardized. SE, Standard Error; CI, Confidence Interval; PSM, Public Service Motivation; SOC-R, Sense of Community Responsibility; SOC, Sense of Community; CR, Community Resilience.

## 4 Conclsion and discussion

### 4.1 Conclusion

In order to understand how individual-level PSM affects residents “assessments of group-level PCR, this study examined the potential impact of PSM, SOC-R, and SOC on PCR using a chain-mediated model. It was found that residents” PSM would influence assessments of PCR, and the chain mediating role of SOC-R and SOC in it is significant. Therefore, all the hypotheses of this study were tested.

### 4.2 Discussion

This study supports the hypothesis of the role of community social capital theory, which suggests that the generation of community social capital requires the involvement of individuals in the group or their willingness to contribute to the group, and that the accumulation of community social capital enhances the resilience of the community as a whole and improves the individual's assessment of PCR. Consistent with previous findings, increased PSM leads to increased SOC-R and SOC (Chen et al., 2023; Jiartana et al., 2023) and the latter two are predictive of CR as community social capital (Gamo and Park, 2024; Mannarini et al., 2022). It has also been shown that the general tendency of PSM translates to some extent into a SOC-R and SOC, which mediates the effect of PSM on employee wellbeing (Boyd and Nowell, 2020). This is similar to the hypothesis of this study, which proposes SOC-R and SOC as mediators of PSM on residents‘ perceived community resilience is also supported by the results. An interesting point is that the effect of general demographic characteristics (e.g., gender, age, and Type of residence) on residents' community resilience assessment was not found in our study, which is different from previous studies, and the reasons for this need to be explored in subsequent studies.

To improve PCR, this study proposes that residents' PSM predicts SOC-R, which in turn predicts SOC, and ultimately predicts residents' assessment of CR. The findings also support our conceptual model. To date, there has been no research on this relationship, i.e., individual PSM through organizational aspects to its effect on community resilience evaluations. As the majority of individuals in a group have a more “other” orientation, this leads to higher expectations of the community's ability to cope with a disaster, which in turn leads to higher evaluations of the resilience of residents in their own neighborhoods. Findings may also provide useful insights for increasing perceived CR, suggesting that when people are motivated to perform public service out of compassion or self-sacrifice, they feel more responsible for their community and want to be involved in their community's affairs (Masukela et al., 2023). This gives the individual a greater sense of belonging to the community, which means that a sense of community will follow. Similarly, individuals become closer to the group (Jetten et al., 2017), which is more conducive to community development thus increasing community resilience. On the other hand, this study is based on residents' subjective evaluation of community resilience, which is also different from community resilience measures based on objective indicators in previous studies.

In practice, based on our findings that PSM positively predicts SOC-R and SOC, which in turn enhance PCR, interventions aimed at fostering residents “public service motivation could be beneficial. Communities could develop programs or environments that encourage and recognize voluntary contributions and prosocial behaviors, thereby nurturing individuals” inherent motivation to serve the public good. As residents become more motivated to engage in community-serving activities, they are likely to develop a stronger sense of responsibility and belonging toward their community. This process contributes to the accumulation of community social capital, which is essential for enhancing collective resilience. Moreover, individuals with higher PSM are more inclined to participate actively in community affairs (Patel et al., 2017), further reinforcing social networks and mutual trust—key components of a resilient community. Overall, cultivating public service motivation among residents not only strengthens their emotional and responsible connections to the community but also builds a solid foundation for sustained community resilience (Song and Wang, 2021).

Further, the practical value of our findings lies in revealing an intervention pathway focused on systematically cultivating community psychological capital by activating residents' intrinsic motivations. Specific strategies—such as designing recruitment materials emphasizing values like “compassionate mutual aid,” “collective commitment to public values,” and “collective efficacy,” or establishing “Community Resilience Ambassador” programs—are not merely procedural. Rather, their significance lies in how they systematically operationalize the transformation from general public service motivation (PSM) to community-specific psychological identifications. This provides real-world validation of the core mechanism identified in this study: by intentionally creating a community environment that advocates contribution and mutual support (through strategic interventions), residents' abstract prosocial motivation (PSM) can be effectively channeled into a sense of responsibility (SOC-R) and belonging (SOC) toward their specific community. This psychological transition from “motivation” to “responsibility” and then to “identification” constitutes the key process through which community social capital is built and PCR is ultimately enhanced. Thus, the practical contribution of this research is to direct community-building efforts beyond mere resource investment toward the systematic cultivation and transformation of residents' intrinsic motivations and psychological attachments, offering a targeted and sustainable psychological pathway to enhance community resilience.

## 5 Research limitations and prospects

This study had several limitations that could be addressed by future research. First, CR is not the same as perceived CR, because it is influenced by individuals' own perceptions. For example, probabilistic perception theory posits that although the environment provides a large number of perceptual clues, only a small proportion of these are useful for observers, because they only pay attention to those clues that are understandable (Brunswik, 1955).

Secondly, the use of cross-sectional data precludes the possibility of drawing causal inferences. Furthermore, the potential influence of confounding variables, such as participants' attitudes toward the community or their status as public officials, was not considered. Consequently, additional longitudinal or experimental studies are required to extend the present findings.

Ultimately, despite the large sample size of this study, which encompassed residents of three Chinese cities, more objective measures of behavior are required to further examine the observed associations between variables. The present findings could inform the design of field experiments to study how these variables affect CR, and provide a basis for future research on CR.

In addition, the PCR measurement tools used in this study do not provide a complete explanation of residents' perceptions of resilience in their own community. We found that residents in the community know little about CR in the community in which they live, regardless of how closely their subjective evaluations are aligned with official, objective measures, or the extent to which they can accurately assess community resilience in the community in which they live. Much of the research on community resilience in current studies has been in the area of risk, and most of the research related to residents' own perceptions of CR has used interviews or participatory research. Future research should focus on quantitative studies of residents' own perceptions of community resilience. And it should not be limited to communities where disasters occur.

## Data Availability

The raw data supporting the conclusions of this article will be made available by the authors, without undue reservation.
